# Women’s perceptions of antenatal care: are we following guideline recommended care?

**DOI:** 10.1186/s12884-016-0984-y

**Published:** 2016-07-27

**Authors:** Amy Waller, Jamie Bryant, Emilie Cameron, Mohamed Galal, Juliana Quay, Rob Sanson-Fisher

**Affiliations:** 1 Priority Research Centre for Health Behaviour, University of Newcastle & Hunter Medical Research Institute, HMRI Building, Callaghan, NSW 2308 Australia; 2 School of Medicine and Public Health, University of Newcastle, Callaghan, NSW Australia; 3 Department of Obstetrics and Gynaecology, Hunter New England Local Health District, Newcastle, NSW Australia; 4 Priority Research Centre for Reproductive Science, University of Newcastle, Callaghan, NSW Australia

**Keywords:** Antenatal, Guidelines, Implementation, Screening, Pregnancy

## Abstract

**Background:**

Detection and management of antenatal risk factors is critical for improved maternal and infant outcomes. This study describes the proportion of pregnant women who self-reported being screened for and offered advice to manage antenatal risk factors in line with antenatal care recommendations; and the characteristics associated with rates of screening.

**Methods:**

A survey was undertaken with 223 (64 % of eligible) pregnant women recruited from an outpatient obstetrics clinic at a public hospital. Participants self-reported whether they were: (i) screened for 23 guideline-recommended risk factors during their antenatal visit; (ii) offered assistance to manage identified risk factor(s); and (iii) received assistance that was of benefit. Association between rate of screening and participant characteristics was examined using multivariate quantile regression.

**Results:**

Overall, 23 % of women reported that they were asked about every risk factor. Weight gain (48 %), diet (60 %) and oral health (31 %) were least frequently screened risk factors. The number of women who reported they were offered advice to manage identified risks and the value of that advice was perceived by women as suboptimal. Those women receiving shared care between a midwife and general practitioner, of Aboriginal or Torres Strait Islander descent, and without private health insurance reported being screened for a greater number of risk factors.

**Conclusions:**

Pregnant women report suboptimal rates of screening and management of antenatal risk factors. Initiatives to improve consistency in detection of antenatal risk factors and the application of appropriate interventions to manage those risk factors that are detected are required.

## Background

A significant gap between best scientific evidence and clinical practice exists across many areas of health care [1]. Previous studies have shown evidence–practice gaps are represented by misuse, underuse or overuse across the health care system [2]. The magnitude of the gap varies across medical conditions, with greater concerns reported in relation to underuse compared to overuse [2]. Developing and implementing strategies that can translate evidence into best scientific practice is critical to reduce evidence-practice gaps [3].

Quality of care in maternal services is defined as “*a minimum level of care to all pregnant women and their newborn babies and a higher level of care to those who need it*” [4]. Disparities in quality of antenatal care have been identified internationally, with low rates of detection of risk factors for poor maternal and perinatal outcomes, including smoking, alcohol consumption, obesity, domestic violence, hypertension and depression reported [5–10]. While evidence-based interventions are available to manage many of these modifiable risk factors [11–13], there are inconsistencies in the application of these interventions for women identified as ‘at-risk’ [14]. This may occur when providers fail to offer appropriate advice, support and/or referrals, or when uptake or adherence to offered treatments is poor [15, 16]. There are also particular sub-groups of vulnerable women for whom interventions may be less effective.

In an attempt to address these disparities, policy and professional organisations have developed antenatal care guidelines that make recommendations about screening across key areas of antenatal care including clinical assessments, maternal and fetal screening and lifestyle behaviours. In Australia, the Australian Health Ministers’ Advisory Council released the *Clinical Practice Guidelines: Antenatal Care* to support clinical decisions about appropriate antenatal care [17]. It is expected that successful implementation of the *ANC Guideline*s will increase the consistency with which risk factors are identified and treated, and subsequently improve the quality of antenatal care received by pregnant women in Australia.

A key step in reducing evidence-practice gaps is to quantify gaps by systematically measuring current practice and comparing this to recommended practice [18]. Use of medical record audit to quantify the detection and management of risk factors allows data to be drawn from a representative sample [19–21]. However, the accuracy of documentation may be questionable and the perceived benefit of treatments cannot always be ascertained [21, 22]. Providers’ perceptions of care may be subject to social desirability reporting and discordant with the views of patients [10, 23]. While there are potential disadvantages to relying on patient perceptions, including recall bias, they offer potentially a more sensitive measure the perceived value of antenatal care delivered [24]. However, no studies have examined the views of pregnant women living in Australia in relation to whether they were screened for the range of guideline-recommended antenatal risk factors, the help that was offered and accepted by those identified as ‘at-risk’, and the perceived benefit of the help that was offered.

## Method

### Objective

To describe the proportion of women who reported that they were: (1) screened for clinical, screening and lifestyle antenatal risk factors by their antenatal care provider during their antenatal visit; (2) offered assistance to manage identified risk factor(s); and (3) that the offered assistance was perceived to be of benefit. Socio-demographic, pregnancy and psychosocial characteristics associated with being screened for antenatal risk factors were also identified.

### Sample

Eligible participants include women who were: currently pregnant, attending the antenatal clinic at a large tertiary public hospital, aged 18 years or older, able to read and understand English, and judged as able to give informed consent and complete the survey. Women needed to have attended at least one previous antenatal visit to ensure an opportunity for screening and offering of assistance by providers.

### Procedure

Eligible women were approached by research staff while waiting for their clinic appointment and invited to participate in the study. Consenting women completed an anonymous survey on a touch screen computer assessing socio-demographic and clinical characteristics, screening and management of risk factors and diet. Only data related to screening and management of risk factors are reported here.

### Measures

#### Development of survey

Potentially relevant items were identified from recommendations described in the ANC Guidelines including: clinical, lifestyle and screening risk factors. The format of items were based on similar surveys of quality of care members of the team have utilised to assess quality of care in oncology populations [25]. Items were reviewed by a panel of behavioural scientists and antenatal care providers until consensus was reached about format and content. Items were then pilot tested with a convenience sample of pregnant women (*n* = 20) and modified based on feedback about comprehension and relevance.

#### Screening and management of risk factors: asked, offered, benefit

Items examined seven lifestyle; eleven clinical and five screening antenatal risk factors described in the *ANC Guidelines*.

- *Lifestyle factors* included: smoking, alcohol intake, recreational drug use, diet, prescription medication, nutritional supplements, oral health.
- *Clinical factors* included: dating ultrasound, weight, height, BMI, weight gain, blood pressure, urine sample, blood sugar, mental health, anxiety and depression, domestic violence
- *Screening factors* included: Down syndrome, HIV, Hepatitis B, Rubella, Syphilis.

For each risk factor, women responded to questions about screening and management at the clinic. First, participants were asked whether a health care worker (i.e., an obstetrician, doctor, midwife or nurse) had screened them for the risk factor e.g., *“Did your health care worker ask you about your smoking status?”* Response options included *“Yes”, “No but I asked about this’, “No”, “Unsure”, “Prefer not to answer”*. Women who responded with either “*Yes” or “No but I asked about this’* were then asked if they were offered any assistance to manage the risk (e.g., *“Yes”, “No because I am not a smoker” “No”)*. Those who answered *“Yes”* were then asked about the type of assistance offered. Examples of assistance were provided for each risk factor. Women were also asked whether the assistance they were offered was of any benefit *(*e.g., *“Yes”, “No”, “Did not accept help” or “For a short time, but I am smoking again”).*

#### Socio-demographic characteristics included

Date of birth; postcode; Aboriginal or Torres Strait Islander status; marital status; private health insurance status, highest level of education, language spoken at home and country of birth.

#### Pregnancy characteristics included

Due date, visits to GP for antenatal care, visits to hospital for antenatal care, weeks of pregnancy at first visit, and number of pregnancies.

#### Model of care

Participants were asked to identify the model of antenatal care they received from one of four options: (1) high risk clinic (i.e., doctor-led care for women with certain medical conditions or complex pregnancies); (2) midwife model (i.e., midwife acting as primary provider of care); (3) shared care model (i.e., general practitioner-led care with hospital visits involving input from midwife or obstetrician) or (4) birth centre (i.e., a team of midwives who provide care). An ‘other’ option was provided.

### Statistical analysis

To examine the representativeness of the sample, the age and gender of eligible responders and those giving birth in NSW [26] were compared using Chi-square analyses. Frequency data were used to describe the assessment and management of each health factor including: (i) the proportion of women who were screened for the risk factor; (ii) the proportion who indicated they needed assistance to manage the risk factor; (iii) the proportion who indicated they needed assistance and were offered it; (iv) the proportion who were offered assistance and accepted that assistance and (v) the proportion who accepted the assistance and found it was of benefit.

An *overall screening adherence score* was calculated by summing those factors women recalled being asked during their antenatal visit. This calculation excluded screening blood glucose levels and conducting a dating ultrasound as these assessments are not recommended for all pregnant women. Therefore the maximum achievable score was 21. Missing responses were replaced with the individual’s average for the remaining questions and those with over 3 missing responses were removed from calculations. To explore predictors of the *overall screening adherence score*, a multivariate quantile regression was performed on the median to account for the skewed outcome measure. The variables included in the regression model were: age, Aboriginal or Torres Strait Islander, country of birth, marital status, education, Socio-Economic Indexes for Areas (SEIFA) decile, with higher scores indicating higher socio-economic status. (Pink B. Socio-economic indexes for areas (SEIFA)–Technical paper. ABS Catalogue no. 2033.0.55.001. Canberra: ABS2011), private health insurance, model of antenatal care, any previous pregnancies, number of GP visits for pregnancy and number of tertiary antenatal visits. The variance inflation factor indicated no multicollinearity between the variables. The regression was conducted as a complete case analysis where those with missing data were excluded. Analyses were conducted in Stata 11.

## Results

### Sample

A total of 347 eligible women attending their second or subsequent antenatal visit were approached to complete the survey. Of these, 223 (64 %) consented to participate. The demographic and pregnancy characteristics of the consenting women are presented in Table 1. The majority of women were in their third trimester (>25 weeks of gestation) when they completed the survey (75 %) and 67 % had had prior pregnancies. On average, participants were 20 weeks pregnant at their first hospital antenatal appointment (SD = 7.1), had five general practitioner (GP) antenatal visits (SD = 3.9) or hospital antenatal visits (SD = 4) prior to completing the survey. Compared to the NSW birthing sample, those who completed the survey were younger (chi square(df4) = 19.0, *p* value = 0.001) and more likely to be born in Australia (chi square =55.7, *p* value < 0.001) [26].

**Table 1:** Demographics and characteristics of pregnancy for sample (*N* = 223)

		Number	Percent	% for NSW in 2012(31)
Age	*under 20*	3	1.4	3.1^a^
	*20–24*	44	20	13
	*25–29*	71	32	27
	*30–34*	69	32	33
	*35–39*	24	11	19
	*over 40*	8	3.7	4.8
Aboriginal or Torres Strait Islander Origin	*Yes*	12	5.4	3.4
	*No*	209	95	97
Marital status	*Married*	106	48	
	*De Facto*	84	38	
	*Never married*	23	10	
	*Divorced or separated*	9	4.1	
Speak English at home	*Yes*	215	97	
	*No*	6	2.7	
Born in Australia	*Yes*	197	89	65
	*No*	24	11	35
Private Health Insurance	*Yes*	68	31	
	*No*	154	69	
Highest level of education	*High School or below*	82	37	
	*Trade or vocational training*	74	33	
	*University degree*	66	30	
Trimester at time of survey	*1*	1	0.5	
	*2*	52	24	
	*3*	166	75	
	*given birth*	2	0.9	
Model of antenatal care	*High risk*	100	46	
	*Midwife*	55	25	
	*Shared care*	56	26	
	*Birth centre*	8	3.6	3.5
Previous pregnancies	*No*	72	33	
	*Yes*	145	67	
Parity	*No births >24 weeks*	89	41	44^b^
	*1*	74	34	33
	*2*	32	15	14
	*3*	17	7.9	5
	*>4*	3	1.4	3.3
Total		223*		98138

^a^NSW data includes those younger than 18 which our study excludes. Comparison was conducted without this category

^b^NSW data counts birth from 20 weeks

*Missing data present so not all factors add to this total

### Women’s self-reported rates of screening for clinical, screening and lifestyle risk factors

Table 2 presents the proportion of women who were asked about each of the clinical, screening and lifestyle risk factors during their antenatal visits. Overall, 23 % of women reported that they were asked about every risk factor in the *ANC Guidelines* (m = 20.4, SD = 3.06, Median = 21, IQR = 18-23, range 9–23). Women were frequently asked or enquired about blood pressure (99 %), smoking status (97 %), prescription medications (96 %), anxiety and depression (94 %) and alcohol intake (92 %). Fewer women reported being asked about oral health (32 %), diet (65 %) and weight gain (56 %). Of the 182 (86 %) women who reported being asked about domestic violence, 176 (99 %) thought that the discussion took place in an appropriate context.

**Table 2:** Proportion of all women who were asked about each of the lifestyle, clinical and screening health factors recommended in the ANC Guidelines (*N* = 217)*

	Asked about N (%)	Patient volunteered N (%)	Never asked N (%)	Unsure N (%)	Prefer not to answer N (%)
Lifestyle factors					
Smoking status	208 (97)	0 (0)	6 (2.8)	1 (0.5)	0 (0)
Prescription medications	206 (96)	0 (0)	5 (2.3)	3 (1.4)	0 (0)
Alcohol intake	197 (92)	0 (0)	15 (7.0)	1 (0.5)	0 (0)
Recreational drug use	190 (89)	0 (0)	21 (10)	2 (0.9)	0 (0)
Nutritional supplements	173 (80)	8 (3.7)	25 (12)	9 (4.2)	0 (0)
Diet	131 (60)	11 (5.1)	60 (28)	14 (6.5)	1 (0.5)
Oral health	67 (31)	3 (1.4)	129 (60)	16 (7.4)	0 (0)
Clinical assessments					
Blood pressure	212 (99)	-	2 (0.9)	1 (0.5)	-
Anxiety and depression	201 (94)	1 (0.5)	12 (5.6)	0 (0)	0 (0)
Dating ultrasound scan	193 (90)	-	18 (8.4)	3 (1.4)	-
Previous, current mental health disorder	189 (88)	0 (0)	22 (10)	3 (1.4)	0 (0)
Domestic violence	182 (86)	0 (0)	29 (14)	0 (0)	1 (0.5)
Blood sugar	177 (84)	-	27 (13)	7 (3.3)	-
Urine analysis	179 (84)	-	28 (13)	7 (3.3)	-
Measure your weight	174 (82)	-	34 (16)	5 (2.4)	-
Measure your height	166 (78)	-	41 (19)	6 (2.8)	-
BMI (body mass index)	108 (51)	-	71 (33)	34 (16)	-
Weight gain	104 (48)	19 (8.8)	83 (39)	9 (4.2)	0 (0)
Screening					
Down syndrome screening	190 (89)	4 (1.9)	16 (7.5)	4 (1.9)	0 (0)
Rubella immunity testing	172 (81)	0 (0)	32 (15)	9 (4.2)	0 (0)
Hepatitis B testing	164 (77)	1 (0.5)	33 (15)	15 (7.0)	0 (0)
HIV testing	155 (72)	2 (0.9)	39 (18)	18 (8.4)	0 (0)
Syphilis testing	152 (71)	0 (0)	35 (16)	27 (12.6)	0 (0)

* Denominators for the proportions were calculated removing missing responses. Minimum *N* = 211

### Women’s self-reported management of clinical, screening and lifestyle antenatal risk factors

#### Did women need help to manage risk factors?

Table 3 presents the proportion of women who indicated that they needed help to manage each of the clinical, screening and lifestyle risk factors. Overall, 70 % (*n* = 156) women reported needing assistance to manage at least one risk factor. Women reported needing help with managing their: weight (35 %), deciding which nutritional supplement to take (33 %), anxiety and depression (26 %) and oral health (25 %). Fewer women reported needing help to manage alcohol intake (5 %), recreational drug use (5 %), prescription medication use (7 %) or domestic violence (9 %).

**Table 3:** Proportion of women reporting needing help with health factors, being offered assistance, accepting help and finding the help beneficial

Health factor	N(%) needing assistance*	N(%) offered assistance*	N(%) accepting assistance*	N(%) finding assistance helpful*
Manage your weight	74 (35)	27 (36)	20 (80)	16 (80)
Safety of nutritional supplements	70 (33)	35 (50)	29 (94)	26 (90)
Help with anxiety and depression	53 (26)	44 (85)	28 (68)	23 (82)
Improve your oral health	53 (25)	7 (13)	5 (100)	4 (80)
Manage your blood sugar levels	50 (24)	45 (90)	37 (95)	35 (95)
Manage an abnormal urine sample	48 (23)	42 (88)	33 (87)	30 (91)
Quit smoking	37 (18)	23 (62)	12 (63)	8 (67)
Help with mental illness	33 (16)	26 (79)	15 (68)	12 (80)
Manage high blood pressure	33 (16)	22 (67)	16 (84)	14 (88)
Help with domestic violence	19 (9)	9 (47)	4 (50)	2 (50)
Stop taking unsafe prescription medications	14 (7)	6 (43)	6 (100)	5 (83)
Stop drinking	10 (5)	1 (10)	1 (100)	0 (0)
Stop taking recreational drugs	11 (5)	3 (27)	3 (100)	2 (67)

* Denominators for the proportions were calculated removing missing responses

#### Were women offered help to manage risk factors?

Table 3 also presents the proportion of women reporting that they needed help to manage risk factors and the proportion that were then offered and accepted help to manage identified risks by providers. Women were more likely to be offered help to manage clinical risk factors such as anxiety and depression (85 %), mental health issues (79 %) and blood sugar levels (90 %). Women who reported needing assistance with managing lifestyle factors, such as weight gain, smoking, nutritional supplements and oral health, were less likely to report that they were offered help. Less than half of women who needed assistance reported they were offered help for domestic violence (47 %), prescription medications (43 %), alcohol intake (10 %) and recreational drugs (27 %), however the number reporting a need for assistance with these issues was small (i.e., all *n* < 20).

#### Did women find the help they were offered beneficial?

Women were less likely to accept assistance for domestic violence (50 %), smoking (63 %), mental illness (68 %) and anxiety and depression (68 %). The proportion of women who reported that the help they were offered was beneficial varied across health factors (Table 3). Fewer women reported that the help they received for domestic violence (50 %), recreational drug use (67 %) and smoking (67 %) was of benefit, however the number reporting they accepted help for these issues was small (i.e., *n* < 12).

### Predictors of overall screening adherence score

The median number of risk factors that women recall discussing with their health care worker during antenatal care was 18 (IQR = 15–20; *N* = 211). The results of the multivariate quantile regression performed on the *overall self*-*reported screening adherence score* are shown in Table 4. Ten percent of the sample were missing at least one demographic variable and were excluded from the complete case analysis. After adjusting for covariates, women who were of Aboriginal or Torres Strait Islander descent and those who did not have private health insurance were asked about significantly more factors. Those who were receiving shared care between the GP and midwife were asked about significantly more risk factors, compared with those attending the midwife clinic alone.

**Table 4:** Predictors of being asked about more health factors (*N* = 189)

Screen score		Number	Median health factors asked about (IQR)	Coefficient (95 % CI)	*P*-value
Aboriginal or Torres Strait Islander Origin	*No*	179	18 (15–20)	ref	ref
	*Yes*	10	20 (17–21)	2.084 (0.147–4.021)	0.035
Born in Australia	*Yes*	172	19 (15–20)	ref	ref
	*No*	17	17 (16–19)	−1.065 (−2.776–0.645)	0.221
Marital status	*Married/ De Facto*	163	18 (15–20)	ref	ref
	*Never married*	20	18 (16–20)	−0.056 (−1.771–1.658)	0.948
	*Divorced or separated*	6	21 (17–21)	−0.386 (−3.202–2.43)	0.787
Highest level of education	*High School or below*	66	18 (15–20)	ref	ref
	*Trade or vocational training*	64	19 (16–20)	0.227 (−1.03–1.484)	0.721
	*University degree*	59	18 (15–19)	0.117 (−1.241–1.476)	0.865
Private Health Insurance	*No*	131	19 (16–20)	ref	ref
	*Yes*	58	17 (15–19)	−1.29 (−2.468–0.112)	0.032
Model of antenatal care	*Midwife*	45	17 (15–20)	ref	ref
	*High risk*	92	18.5 (16–20)	0.888 (−0.446–2.222)	0.191
	*Shared care*	45	19 (16–20)	1.699 (0.202–3.197)	0.026
	*Birth centre*	7	18 (15–19)	0.692 (−2.137–3.522)	0.63
Previous pregnancies	*Yes*	127	18 (15–20)	ref	ref
	*No*	62	19 (16–20)	0.552 (−0.6–1.705)	0.346
Age				0.048 (−0.058–0.154)	0.373
Weeks pregnant					
Number of visits to GP for antenatal care				−0.021 (−0.134–0.091)	0.707
Number of tertiary antenatal visits				0.083 (−0.039–0.205)	0.18
SEIFA				0.007 (−0.004–0.017)	0.232
Constant				9.23 (−1.542–20.001)	0.093

## Discussion

An apparent deficit by antenatal care providers to undertake routine screening or treatment for known antenatal risk factors and complications may contribute to potentially avoidable adverse maternal and fetal outcomes [27–29]. This is the first study to describe the rate of screening and treatment of risk factors as recommended in the Australian Health Ministers’ Advisory Council *Clinical Practice Guidelines: Antenatal Care* from the perspective of pregnant women.

Antenatal visits are seen to represent a potentially powerful “teachable moment” for the promotion of healthy behaviours and the avoidance of adverse maternal and infant outcomes. Positive associations between adherence to recommendations and perinatal outcomes; and between the number of health promotion topics discussed and health behaviours during pregnancy have been reported [30, 31]. It is therefore promising that women in this study were frequently screened for risk factors such as smoking, alcohol intake, recreational drug use and domestic violence. However, less than a quarter of women self-reported that they had been screened for every risk factor. This is consistent with previous international studies. For example, approximately half of the women had <80 % of recommended ANC content documented in a Malaysian study [32]; while a USA study found that less than a third of women had >80 % of all recommended care documented [7].

Diet and weight gain during pregnancy and oral hygiene were relatively under-detected [13, 33]. The breakdown in care at the point of detection is surprising given the increased use of forms in medical records that are used by providers in some antenatal clinics [22]. Further, the over-representation of women in their third trimester in this sample, with an average of five antenatal visits, suggests that there were multiple opportunities to screen for these risk factors. However, these findings are consistent with previous studies undertaken in both developing and developed countries, which report lower compliance for recommendations about health education and lifestyle behaviours compared to clinical and screening recommendations [5, 34].

Initiatives that have been successful in improving the accuracy and consistency of detection of health risks in other care settings, including primary care and oncology, offer a potential solution [35, 36]. Such initiatives include systematic self-report assessments that can be completed by patients prior to visits with results reported directly to providers [35, 37, 38]; and patient-driven initiatives such as question prompt lists, which may increase the frequency of question-asking by women and initiation of issues that providers may be reluctant or less skilled in raising [24, 39]. Systematic reviews have identified a lack of time and relevant training as common barriers to detection of risk factors that are more likely to go undetected, such as poor nutrition and excessive weight gain [40–42]. Ongoing education and training for providers is needed, given the potentially adverse impact of undetected risks on maternal and fetal outcomes.

There were occasions where risk factors were identified but not managed well. Only two thirds of women in this study who indicated a desire for help to quit smoking report that they were offered this help, despite evidence for the effectiveness of smoking cessation interventions during pregnancy [11, 43]. Strategies that can be implemented with minimal burden and facilitate point-of-care access to evidence based interventions to manage identified risks are critical. For example, providing point-of-care feedback to women and antenatal providers has the potential to guide visits and facilitate opportunistic intervention. Audit and feedback, reminders and multi-faceted approaches have also been shown to be particularly effective in obstetrics settings [29].

Less than half of women who indicated a need for help with domestic violence recalled being offered assistance by their providers. Misconceptions about risk factors, signs and symptoms, as well as a lack of time, training and effective referral pathways may contribute to poor detection and management of domestic violence [44]. These findings highlight the need for strategies to overcome identified barriers. Potential strategies might include: providing women with information about services that are available to help them if experiencing domestic violence (e.g., wallet-sized cards listing appropriate agencies, particularly refugees and sources of legal advice); providing education and training to antenatal care providers to aid them in discussing and responding to domestic violence; providing clear referral pathways and information about local services; enhancing collaboration between domestic violence agencies and antenatal care services (e.g.,., outreach with DV services providing in-service education and training) [44–46]. Ongoing education and training has been moderately successful in improving provider detection of domestic violence [45, 47]. A recent Cochrane review has highlighted the need for further work in developing and testing programs to effectively prevent or reduce domestic violence [48]. As women may be reluctant to discuss issues again if a disclosure is mismanaged [24], this is an important gap that requires further examination.

Women also reported that they did not accept the help offered or did not find the help beneficial. While the numbers were small, this may have been due to the type of assistance offered or their readiness to change risk behaviour. For example, only 63 % of women offered help to quit smoking accepted help. Similarly, of those requiring assistance for anxiety and depression, 59 % were offered a referral to a mental health specialist, however 14 % did not accept the referral. Studies in primary care populations suggest that patient views about the acceptability of psychological treatment is a key factor in poor uptake. People who receive treatment that matches their preference recover more quickly than those who perceive a mismatch between their preferred and received treatment [49]. Therefore, information about patients’ perceived needs and preferences for treatment are likely to be an important adjunct to information on severity of the risk factor. Even when interventions are accepted, desired outcomes or behaviours may not always be achieved. Monitoring risk factors over time may highlight those areas where previous intervention efforts are unsuccessful. The application of higher-intensity interventions tailored to individual needs may be required. ‘Stepped-care’ approaches have had some success in the treatment of risk factors such as depression [50].

This study identified sub-groups of women who were more likely to be screened for a greater number of risk factors. Women of Aboriginal and Torres Strait Islander descent were asked about more risk factors. While this is in contrast to previous studies which suggest this group are less likely to have issues identified [19], this may reflect an increased awareness of the adverse outcomes experienced in this population [51] among the treating team and subsequently concentrated effort to identify risk factors. Whether this identification of risk factors then translates into appropriate management for Indigenous women deemed at-risk requires further examination in a larger sample. Those without private health insurance were also asked about a greater number of health factors. Women who received shared care between the GP and midwife were asked about a greater number of health factors, suggesting an important role for GPs in maternity care [52]. The higher adherence rate reported by women receiving shared care may be due to a greater number of opportunities to be screened by multiple providers. It may also reflect individual-provider or clinic-level variation. Examination of variation at this level requires replication in multiple antenatal clinics and hospitals. The overall screening adherence score did not capture whether there were particular sub-groups who were more likely to be offered help or those who perceived the help was of greater benefit. Previous studies have shown that there may be variability in the follow-up of identified risk factors for some sub-groups [53]; and particular sub-groups for whom interventions are less effective [54–56].

### Limitations

This study was undertaken in a public hospital in Australia, with both high and low risk births and varied models of care. Adherence to recommendations in these guidelines should be examined in other antenatal care settings and with larger samples, to examine potential individual provider- and site-level variations in quality of care. Generalisation of these findings to other countries is cautioned, given that models of care and recommendations regarding the management of antenatal risk factors vary. For example, a recent systematic review of existing antenatal care (ANC) guidelines identified 15 routine ANC guidelines and 70 relating to specific situations or risk factors [57]. Of the 171 recommendations made in these guidelines, inconsistencies were reported for 33 recommendations [57]. There was also an over-representation of younger and Australian-born women in the sample. Women of non-English speaking background in developed countries such as Australia report higher unmet needs, lower satisfaction with care, suboptimal communication and health literacy and experience poorer perinatal outcomes [58]. Targeted approaches to quality improvement should be informed by perceived quality of care from the perspective of this vulnerable sub-group. We also did not examine the offering of assistance to those women who did not need it (e.g., provision of quitting advice to a non-smoker). Receipt of unnecessary health services may also contribute to evidence-practice gaps in health care and should be examined in the context of antenatal care [31]. There may also have been disparities between actual performance and women’s self-reported performance. For example, women may under-report the need for assistance in some areas, such as alcohol intake or domestic violence. Integrating multiple data methods, including self-report, medical record audits and/or observation should be considered in future studies to confirm findings reflect actual variation rather than patient recall.

### Conclusion

Pregnant women report suboptimal rates of screening and management of antenatal risk factors. Initiatives to improve consistency in detection of antenatal risk factors and the application of appropriate interventions to manage those risk factors that are detected are required. Quality improvement approaches involving systematic monitoring of risk factors combined with feedback to health care providers have been implemented in a range of care settings to reduce evidence-practice gaps and promote timely intervention. Such initiatives hold promise in antenatal care settings, however prospective identification of barriers and enablers to change is needed to help facilitate the success of such initiatives.
